# Social differences between the general population and tuberculosis patients in Slovakia

**DOI:** 10.3389/fpubh.2025.1541882

**Published:** 2025-02-28

**Authors:** Peter Vyšehradský, Ivan Solovič, Lucia Kotúľová, Marián Grendár, Monika Rákošová, Henrieta Hudečková, Robert Vyšehradský

**Affiliations:** ^1^Institute of Public Health, Jessenius Faculty of Medicine in Martin, Comenius University in Bratislava, Martin, Slovakia; ^2^National Institute of Tuberculosis, Lung Diseases and Thoracic Surgery, Vyšné Hágy, Slovakia; ^3^Biomedical Centre Martin, Jessenius Faculty of Medicine in Martin, Comenius University in Bratislava, Martin, Slovakia; ^4^Statistical Office of the Slovak Republic, Martin, Slovakia; ^5^Clinic of Pneumology and Phthisiology, Jessenius Faculty of Medicine in Martin, Comenius University in Bratislava, Martin, Slovakia

**Keywords:** tuberculosis, education, poverty, smoking, alcohol

## Abstract

**Objectives:**

Point out the social specifics of patients with tuberculosis, which persist despite the socioeconomic development of Slovak society.

**Methods:**

A questionnaire survey was conducted on a sample of consecutive patients with newly diagnosed tuberculosis during the first half of 2023. The collected data were aggregated and compared with the results of European Health Interview Survey (EHIS) in 2019.

**Results:**

We found significant differences in the distribution of the level of education, labour status, nutritional and marital status, financial poverty, smoking habits, alcohol consumption and number of rooms in the dwelling between the general population and TB patients in Slovakia. Moreover, a significant trend in the proportions was observed across contingency table categories for all ordinal variables with more than two levels.

**Conclusion:**

There are several significant social differences between patients with tuberculosis and the general Slovak population.

## 1 Introduction

Tuberculosis has traditionally been considered a disease with significant social determinants. The social wealth and income inequality accounts for approximately 50% of tuberculosis incidence throughout Europe (1). Social status is associated with exposure to infection; influences progression to overt disease; contributes to delays in diagnosis and treatment; and is a predictor of adherence to treatment and treatment success (2). In 2022, an estimated 2.2 million incident cases of TB globally were attributable to malnutrition, 0.73 million to alcohol use disorders, and 0.70 million to smoking (3).

There has been a gradual decrease in the number of notified cases in Slovakia since the second half of the 20th century. The incidence of tuberculosis in Slovakia in 2022 was 2.86/100,000 inhabitants, which is below the long-term average of the European Union (4). Worldwide, TB incidences have declined faster in countries with a high human development index (HDI) (5). Slovakia is currently among the countries with a very high HDI (6). In the past 30 years, Slovak society has undergone significant changes. This started with the revolution in 1989. The impact of the aforementioned social changes on the social status of the population has been evidenced by a significant average annual increase in the HDI in the years 1990–2021 (0.66) (6). However, even in countries without a lack of resources, social factors still determine the incidence of tuberculosis (7). In recent years, the development of the epidemiological situation in Slovakia may have been affected by increasing immigration and the geographically close war conflict. In the years 2019–2021, a drop in HDI was recorded in Slovakia from 0.862 to 0.848. These factors led us to carry out a cross-sectional study focused on the current social situation of Slovak patients with tuberculosis.

## 2 Materials and methods

We conducted a questionnaire survey in patients with newly diagnosed pulmonary or extrapulmonary tuberculosis. There were 102 consecutive patients (33 women) hospitalised at the Slovak National Institute of Tuberculosis, Lung Diseases, and Thoracic Surgery from 1st January to 1st September 2023. With the consent of the Statistical Office of the Slovak Republic, the questionnaire questions were taken from Eurostat European Health Interview Survey (EHIS). The original versions of the questions are available online on the Eurostat website (8). The questionnaire was created in the Google Forms environment. Answers were entered in real time into the form by the interviewer, who was a doctor or nurse. Participation was conditional on the participant giving written informed consent. The survey was approved by the Ethics Committee of the Jessenius Faculty of Medicine in Martin, Comenius University in Bratislava, protocol number 63/2022.

The data obtained by us were aggregated in the same way as the data of the EHIS 2019 survey. The EHIS 2019 survey in the Slovak Republic was conducted on a sample of 7,480 people, of whom 5,597 people completed the questionnaire. Subsequently, the aggregated data of tuberculosis patients were compared with the data of the Slovak population in order to identify social factors that significantly differ in tuberculosis patients from the rest of the Slovak population. For statistical analysis, the R programming language (version 4.4.2) was used, along with the libraries cited in the References section (9–12). Data from tuberculosis patients (TB) and the general Slovak population (SK EHIS 2019) were analysed across several ordinal variables, such as education, nutritional status, number of rooms in the dwelling, smoking habits, frequency of alcohol consumption and heavy episodic drinking, as well as nominal variables, including labour status and marital status. Pearson’s Chi-Squared Test was used to evaluate whether the distributions of categorical variables differed significantly between the TB and SR EHIS 2019 groups. For ordinal variables with more than two levels, the Cochran-Armitage Test for Trend in Contingency Tables was applied. Additionally, mosaic plots were created for these ordinal variables to visually represent the proportional distributions between the two groups across categories. The significance of the interdependence of individual social factors was tested using the Pearson’s Chi-Squared Test of independence.

## 3 Results

All respondents filled in the questionnaire: we did not record a single refusal to participate.

**Figure 1:**
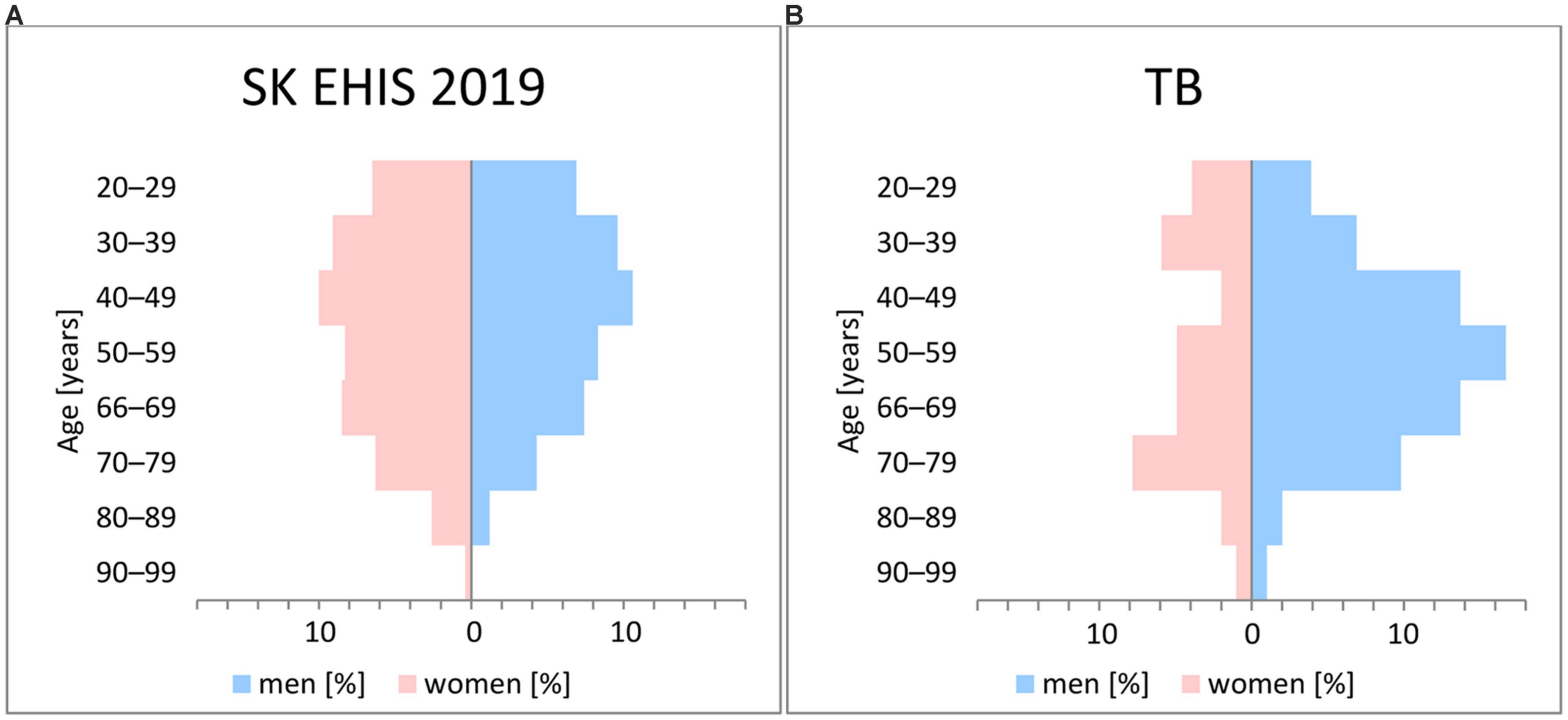
Characteristics of the population. **(A)** General population; **(B)** Tuberculosis patients.

There was a significant difference between the age structure of our group and the base population (*p* < 0.05; tested by Pearson’s Chi-Squared Test of independence). Likewise, the difference in gender distribution (Figure 1) between these sets was statistically significant (*p* < 0.001).

### 3.1 Highest level of education completed

**Figure 2:**
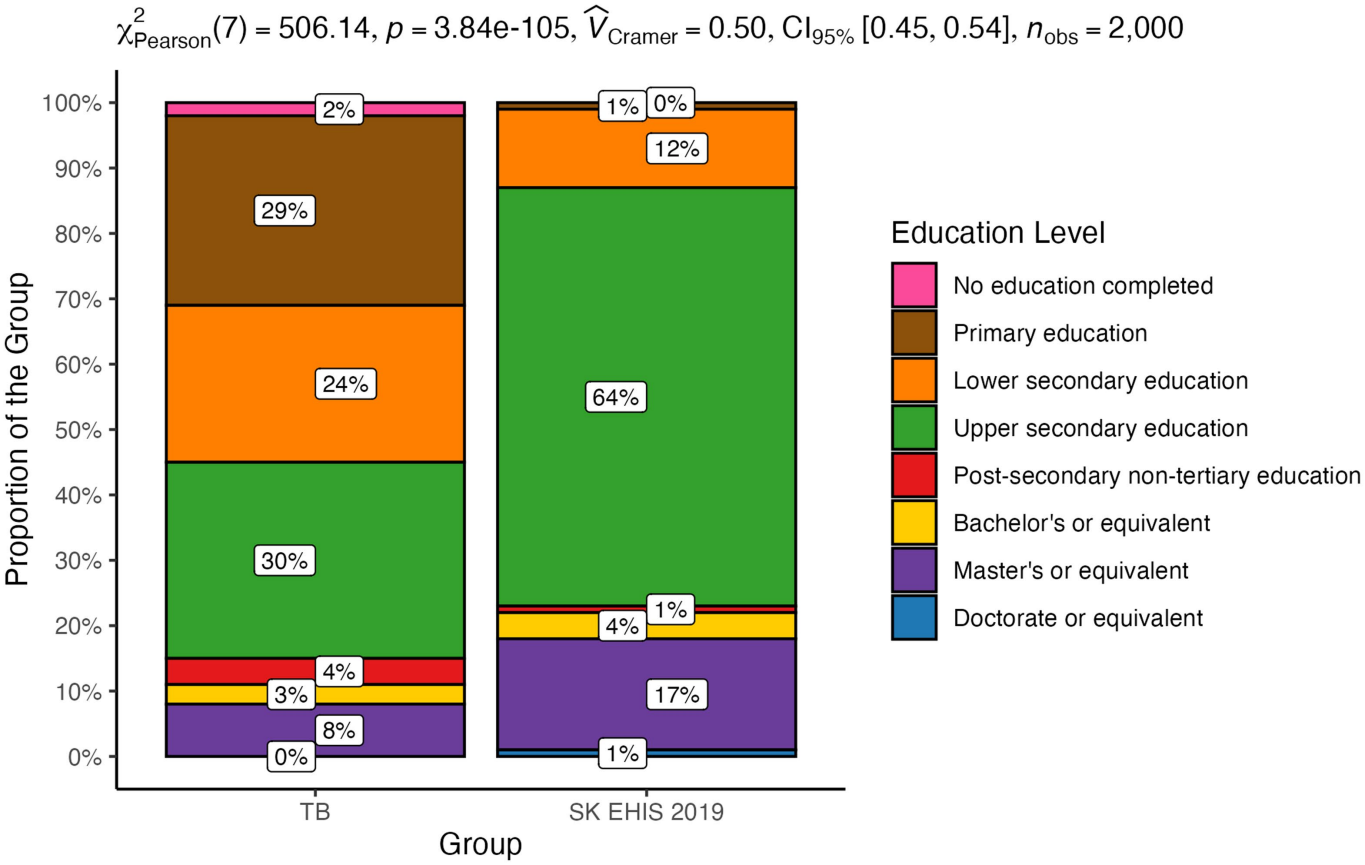
Highest level of education completed—distribution by group.

The proportion of individuals at each education level is shown in Figure 2.

The distribution of education levels between the TB and the SK EHIS 2019 group was significantly different (*p* < 0.0001). This result indicates that individuals with TB are distributed across education levels in a way that is distinct from the SK EHIS 2019 group. This difference is not due to random chance but reflects a real and meaningful disparity between the two groups.

**Figure 3:**
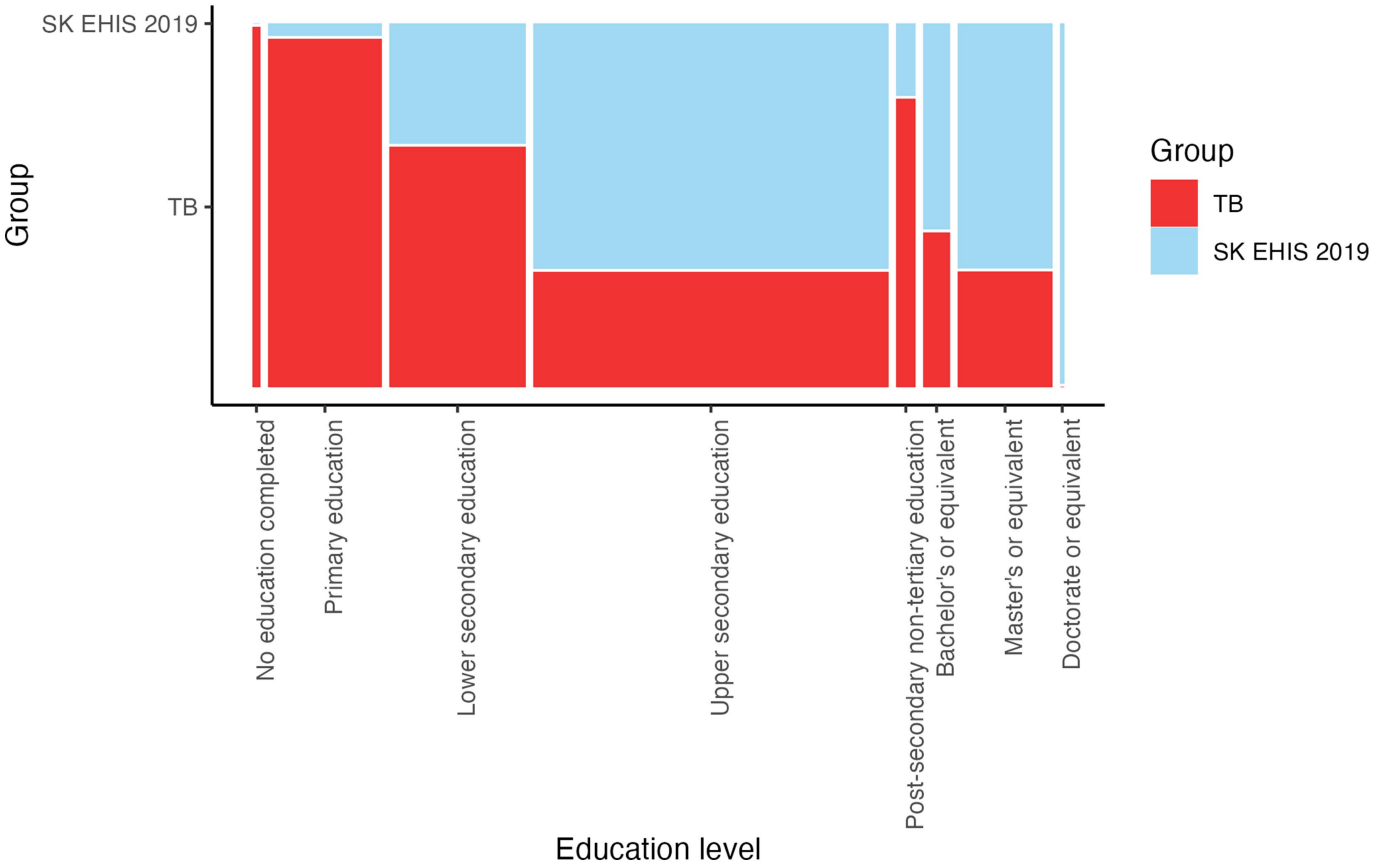
Highest level of education completed—mosaic plot.

The Cochran-Armitage test for trend in contingency tables showed a significant result (*p* < 0.0001). The data reveals that the proportions of TB cases relative to the general Slovak population vary significantly across education levels. From the mosaic plot, we can observe that as education levels increase, the proportion of TB cases tends to decrease, while the proportion in the general population increases (Figure 3).

### 3.2 Labour status

**Figure 4:**
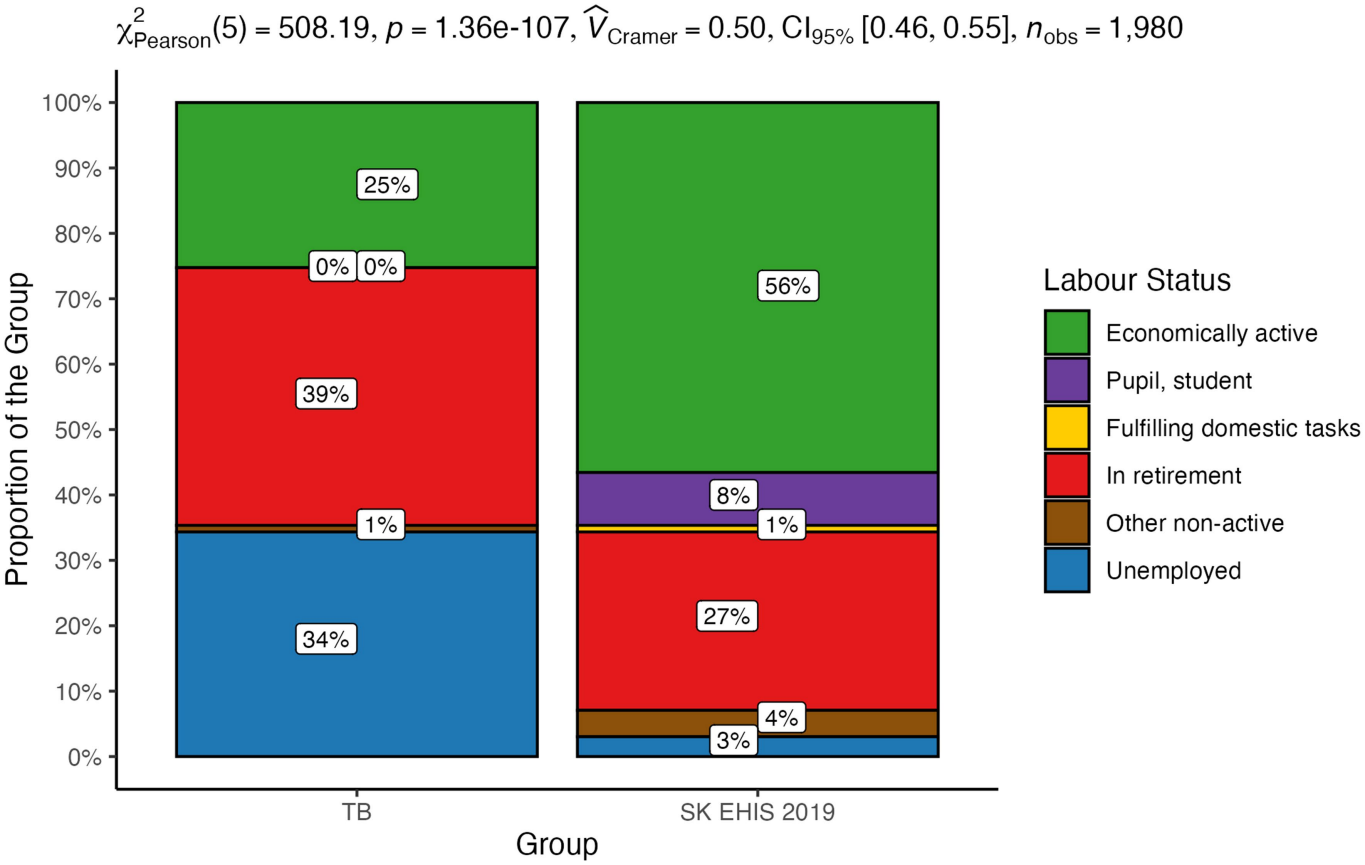
Labour status—distribution by group.

The distribution of labour status categories between the TB group and the SK EHIS 2019 group was significantly different (Figure 4; *p* < 0.0001).

### 3.3 Nutritional status

**Figure 5:**
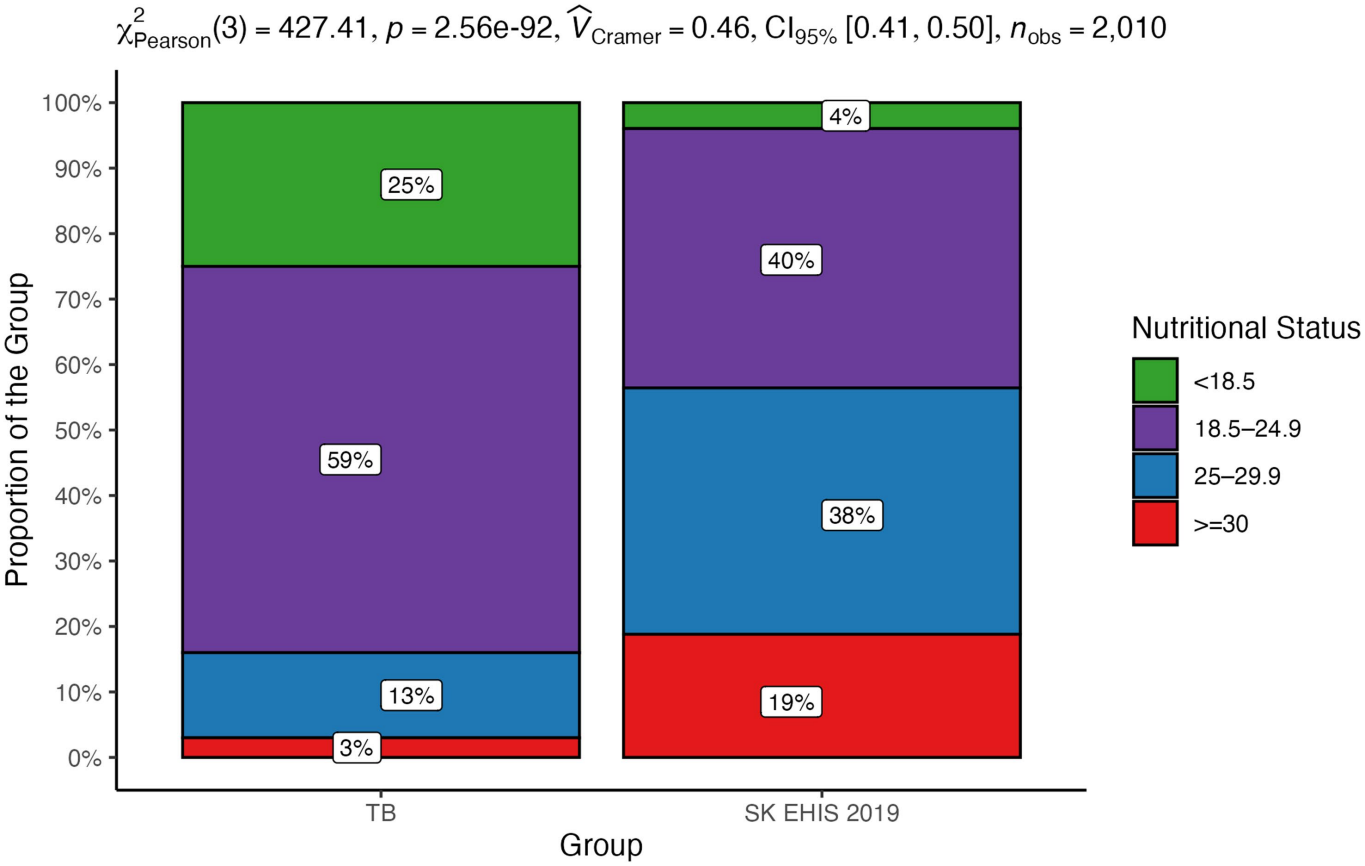
Nutritional status—distribution by group.

Nutritional status was stratified into the following categories: body mass index (BMI) < 18.5 [kg/m^2^]; BMI ≥ 18.5 and <25 [kg/m^2^]; BMI ≥ 25 and <30 [kg/m^2^]; BMI ≥ 30 [kg/m^2^]. The distribution of nutritional status categories between the TB group and the SK EHIS 2019 group was significantly different (Figure 5; *p* < 0.0001).

**Figure 6:**
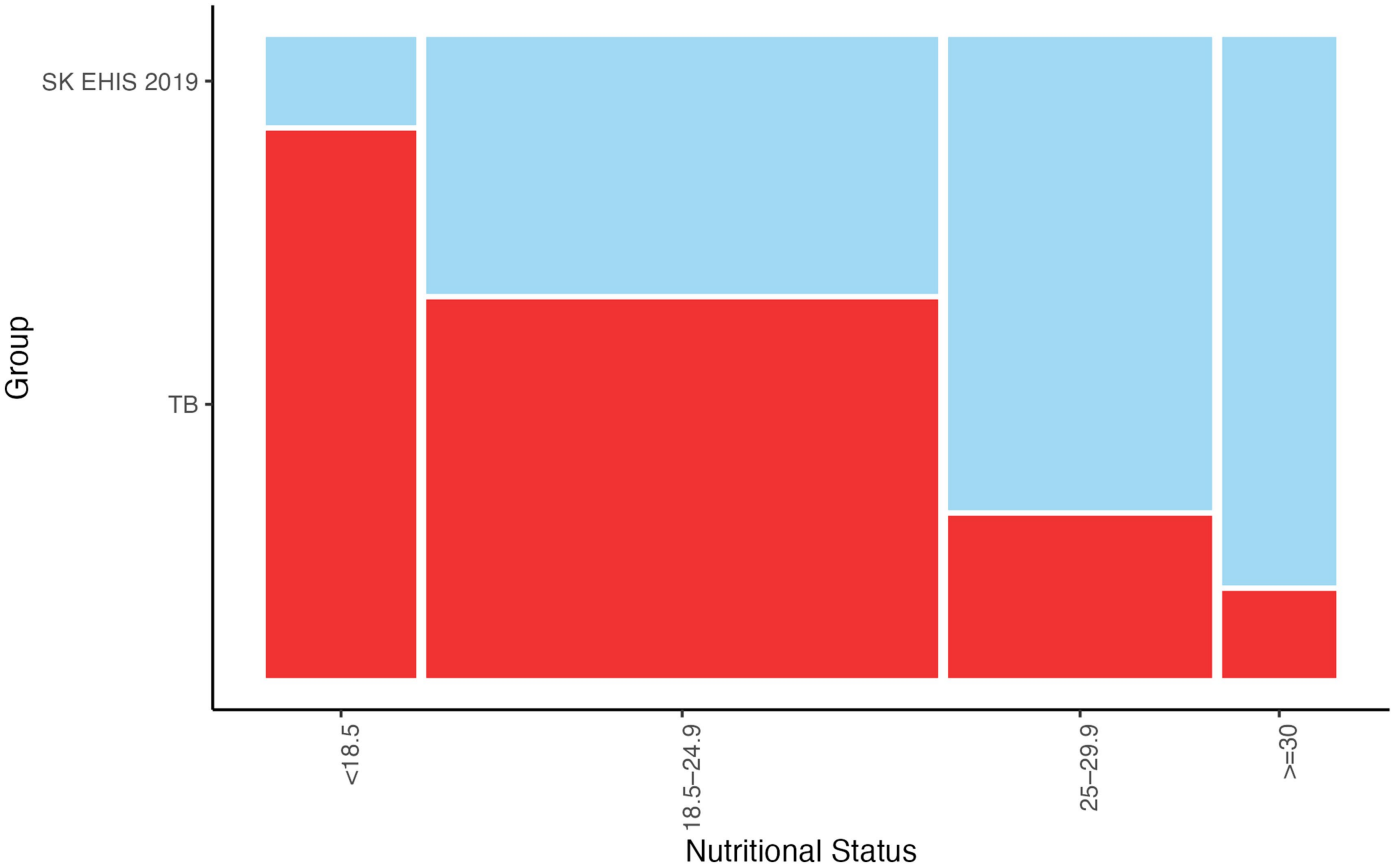
Nutritional status—mosaic plot.

The mosaic plot (Figure 6) shows a decrease in TB case proportions as nutritional status categories progress from BMI < 18.5 [kg/m^2^] to BMI ≥ 30 [kg/m^2^], consistent with the significant trend identified by the Cochran-Armitage test (*p* < 0.0001).

### 3.4 Self-reported income per household member

**Figure 7:**
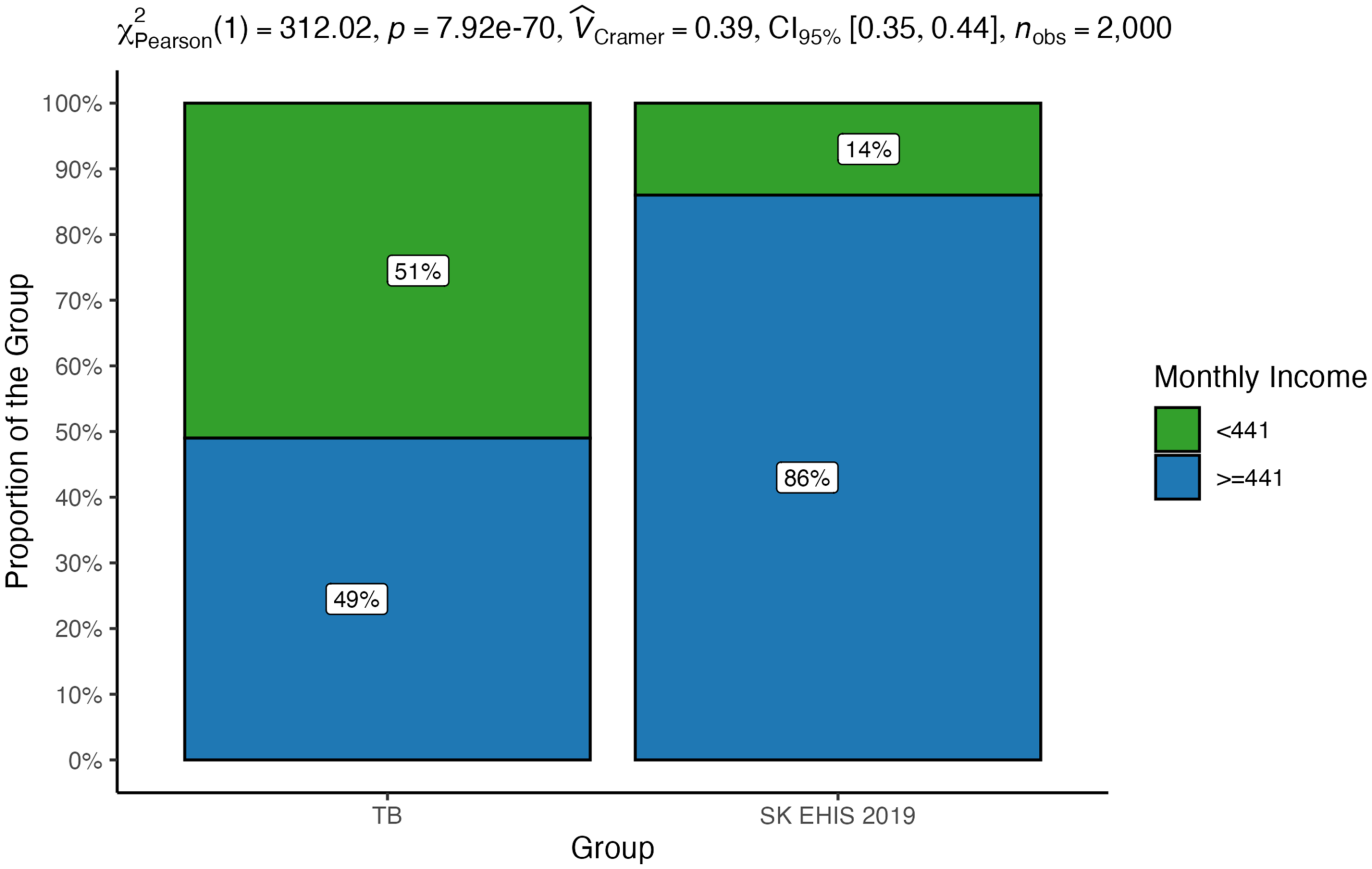
Self-reported income per household member—distribution by group.

The financial poverty line for Slovakia in 2023 was calculated at €441 per month, therefore we calculated the proportion of people below this line in both groups. Self-reported income per household member below financial poverty line was registered more often in patients with tuberculosis. The distribution of monthly income categories between the TB group and the SK EHIS 2019 group was significantly different (Figure 7; *p* < 0.0001).

### 3.5 Smoking habits

**Figure 8:**
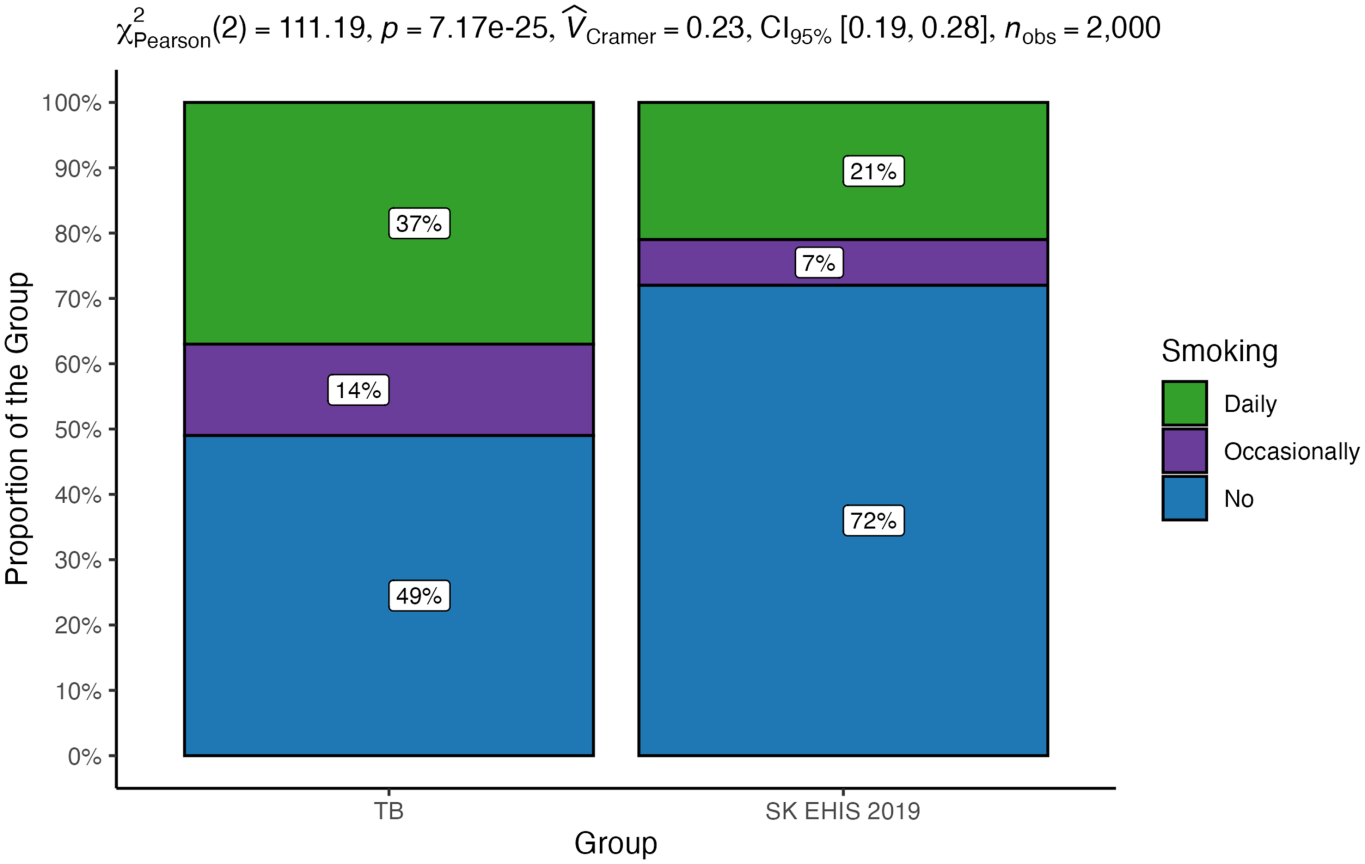
Smoking habits—distribution by group.

The distribution of smoking categories between the TB group and the SK EHIS 2019 group was significantly different (Figure 8; *p* < 0.0001), and the Cochran-Armitage test for trend in contingency tables also showed a significant result (*p* < 0.0001).

**Figure 9:**
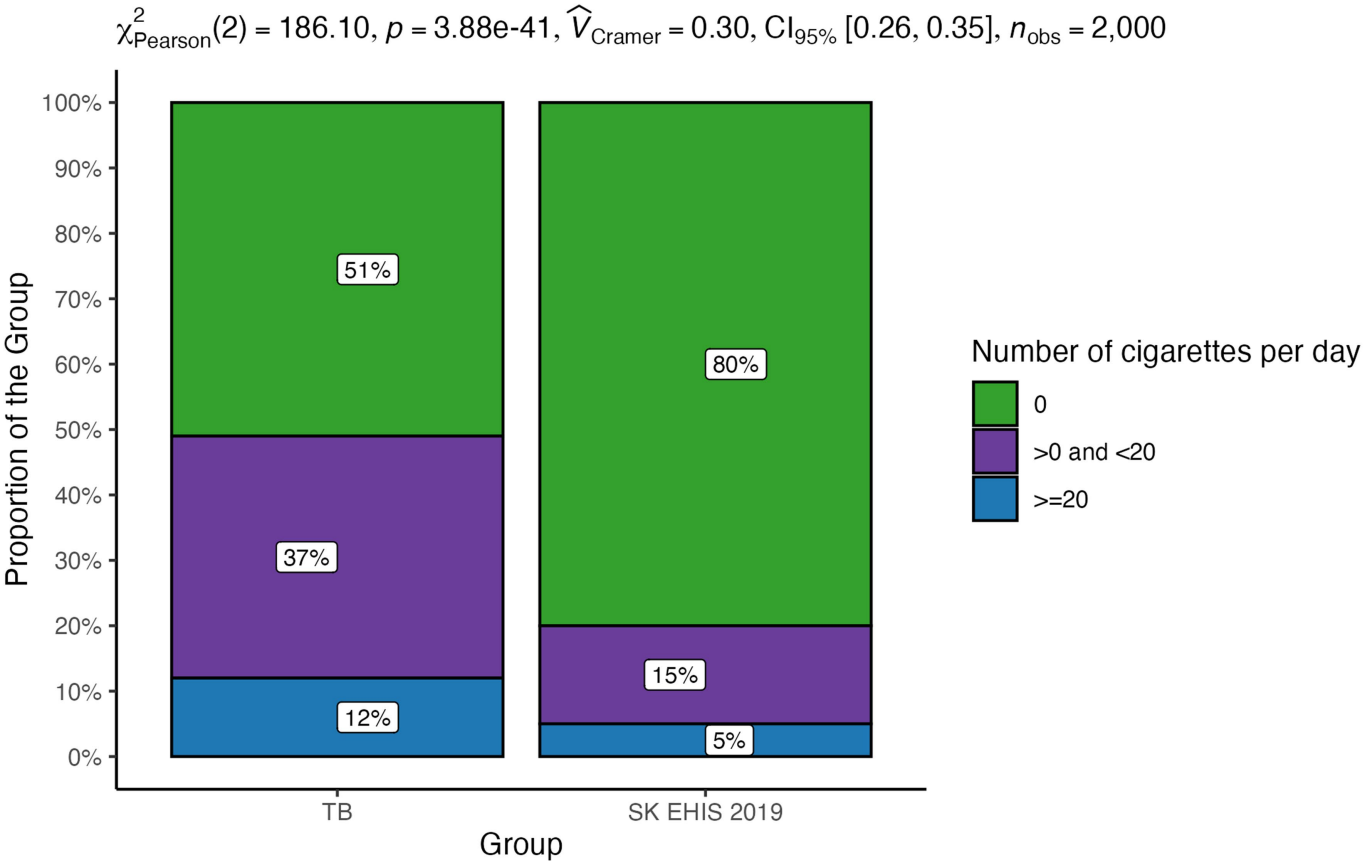
Number of cigarettes per day—distribution by group.

The distribution of the number of cigarettes per day categories between the TB group and the SK EHIS 2019 group differed significantly (Figure 9; *p* < 0.0001), as confirmed by the significant result of the Cochran-Armitage test for trend in contingency tables (*p* < 0.0001).

**Figure 10:**
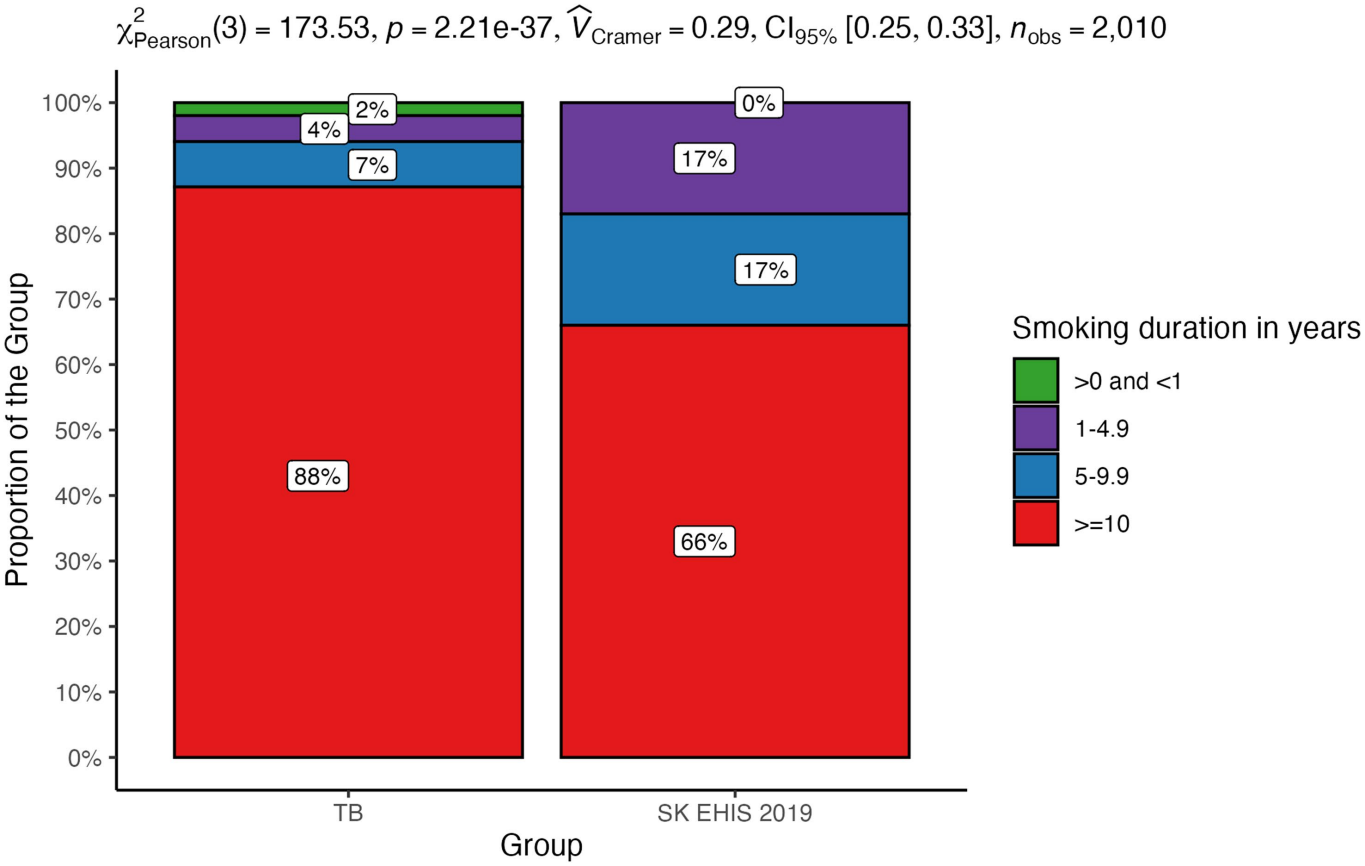
Smoking duration—distribution by group.

The distribution of smoking duration categories between the TB group and the SK EHIS 2019 group differed significantly (Figure 10; *p* < 0.0001), with the Cochran-Armitage test confirming a significant trend (*p* < 0.01) in a contingency table.

**Figure 11:**
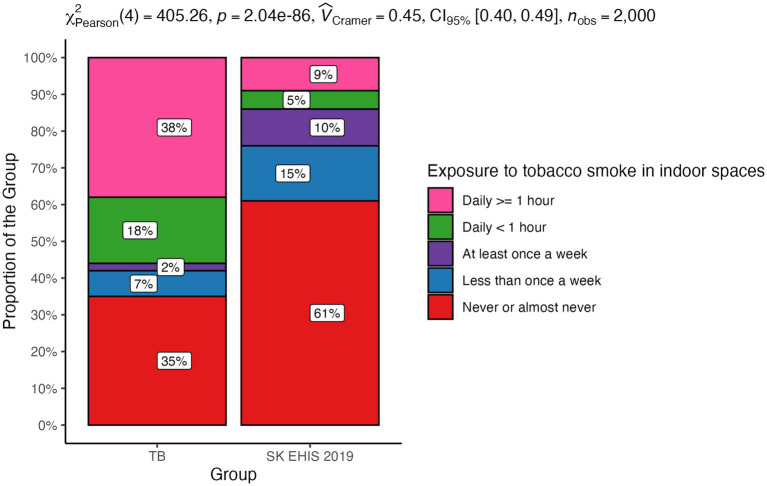
Exposure to tobacco smoke in indoor spaces—distribution by group.

The distribution of exposure to tobacco smoke in indoor spaces categories differed significantly between the TB group and the SK EHIS 2019 group (*p* < 0.0001), with the Cochran-Armitage test showing a significant result (Figure 11; *p* < 0.0001).

### 3.6 Number of rooms in the dwelling

**Figure 12:**
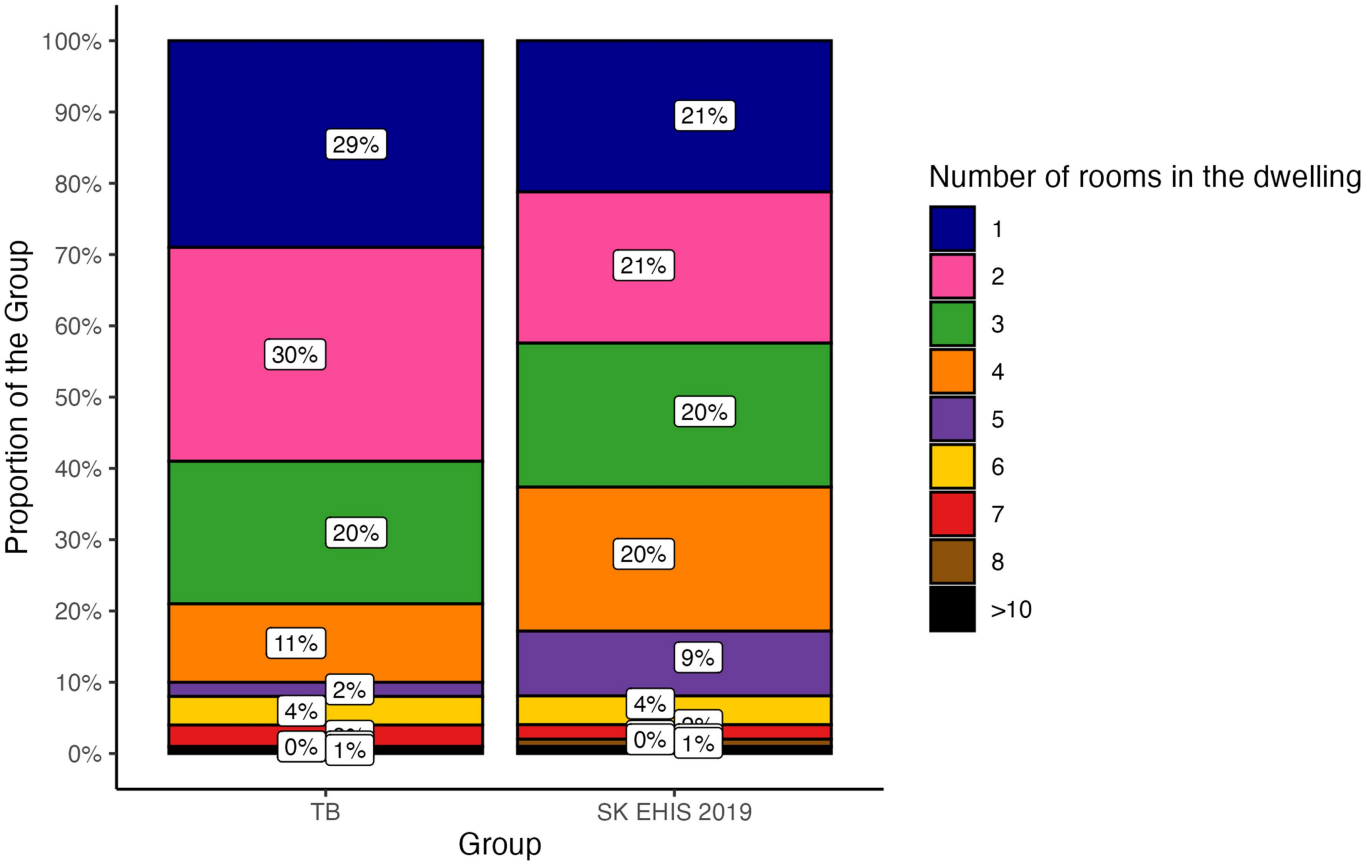
Number of rooms in the dwelling—distribution by group.

The distribution of dwelling room categories differed significantly between the TB group and the SK EHIS 2019 group (Figure 12; *p* < 0.05), with the Cochran-Armitage test confirming a significant result (*p* < 0.01).

### 3.7 Frequency of alcohol consumption

**Figure 13:**
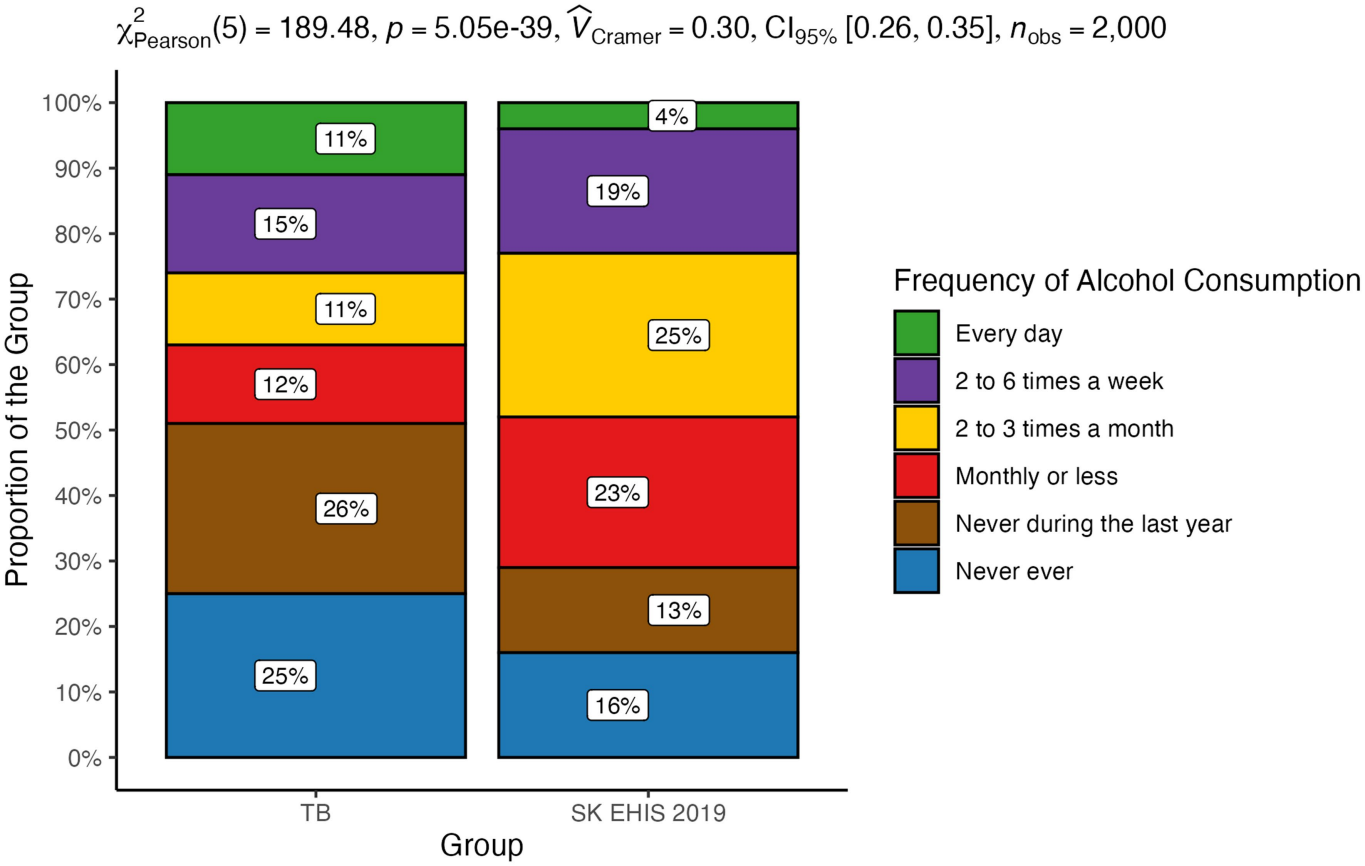
Frequency of alcohol consumption—distribution by group.

Alcohol consumption frequency categories showed a significant disparity between the TB group and the SK EHIS 2019 group (Figure 13; *p* < 0.0001), supported by the significant result of the Cochran-Armitage test (*p* < 0.01).

### 3.8 Heavy episodic drinking

**Figure 14:**
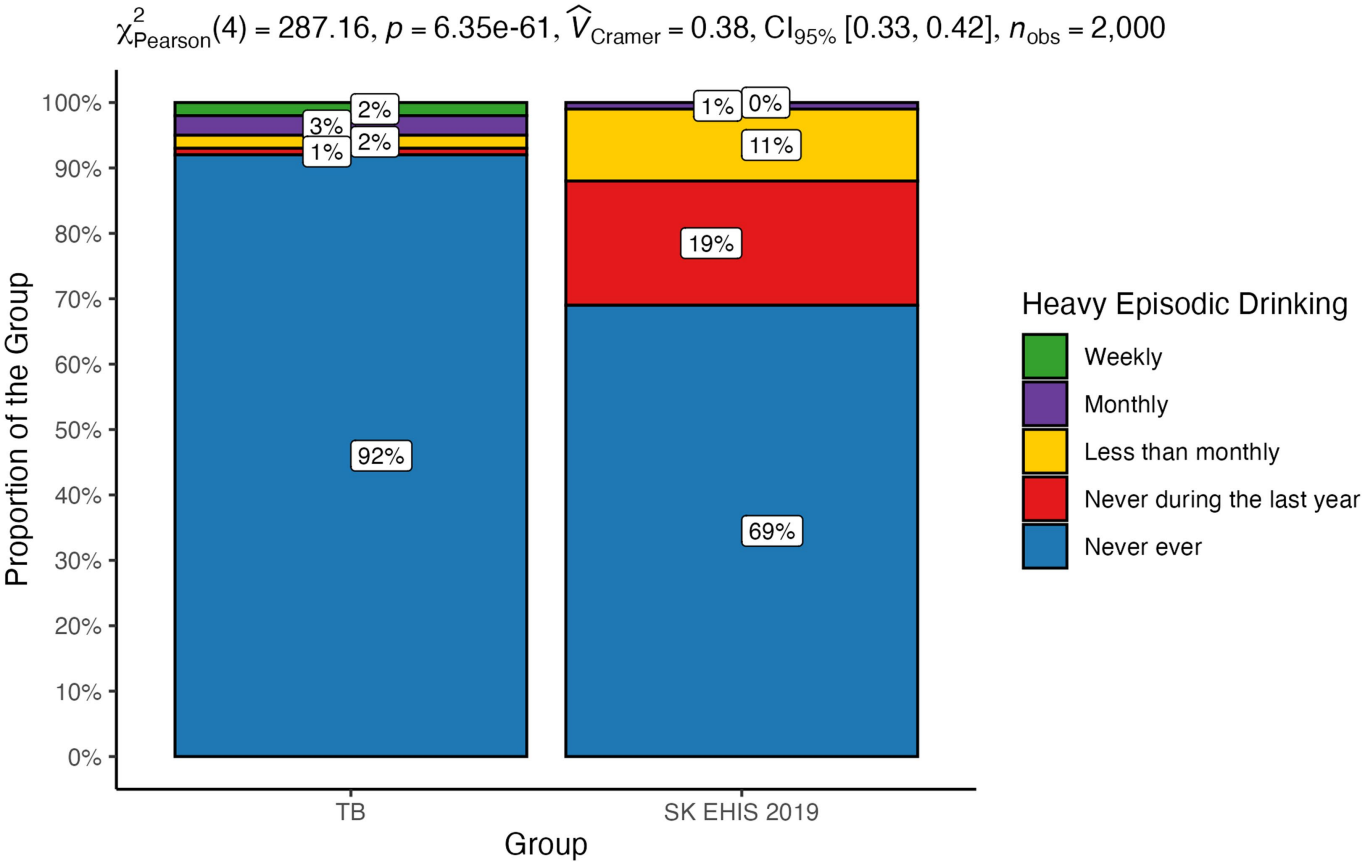
Heavy episodic drinking—distribution by group.

The distribution of heavy episodic drinking categories differed significantly between the TB group and the SK EHIS 2019 group (Figure 14; *p* < 0.0001). The Cochran-Armitage test showed a significant result (*p* < 0.01), revealing a clear decreasing trend in TB case proportions as heavy episodic drinking categories progressed from Weekly to Never during the last year.

### 3.9 Marital status

**Figure 15:**
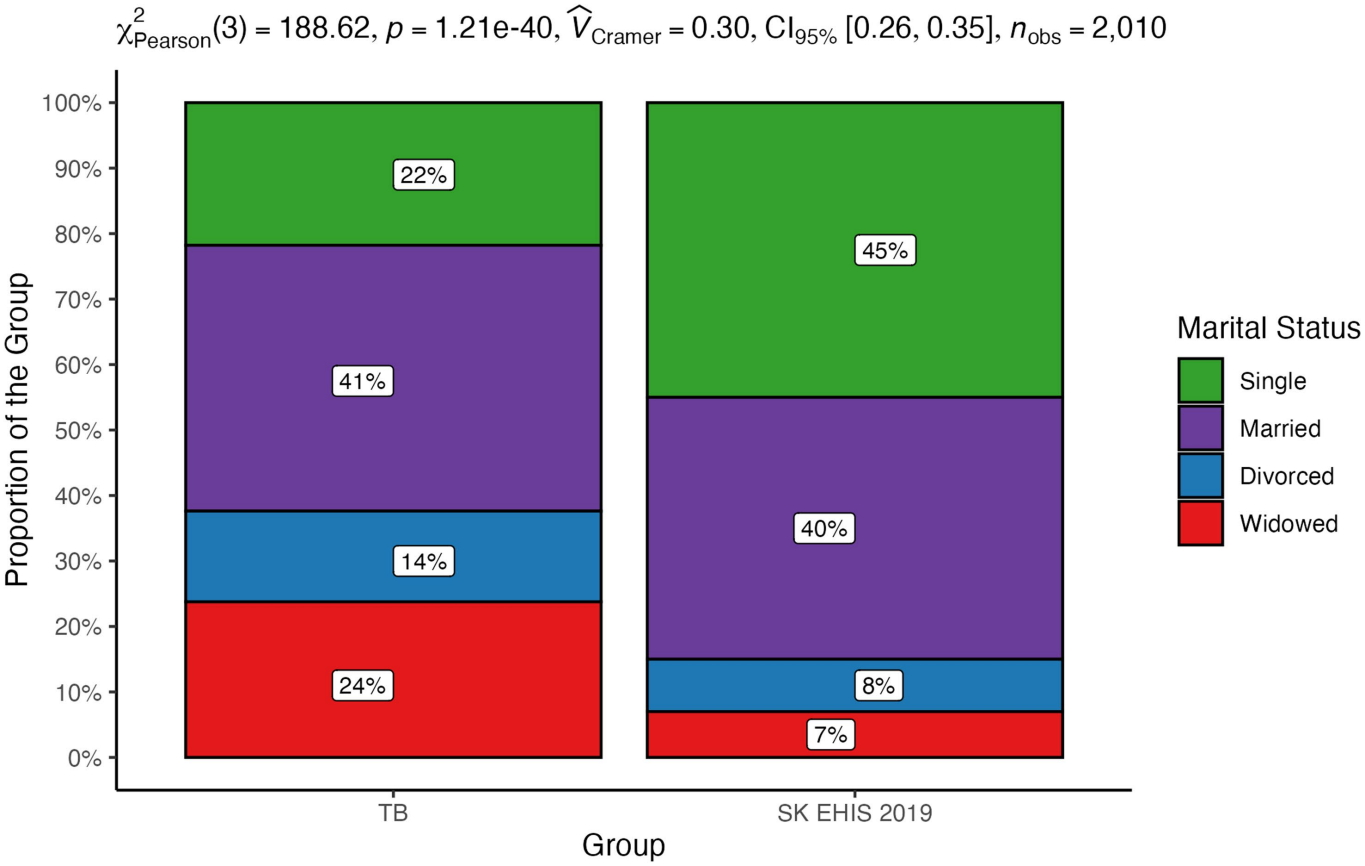
Marital status—distribution by group.

The distribution of marital status categories differed significantly between the TB group and the SK EHIS 2019 group (Figure 15; *p* < 0.0001), reflecting a meaningful disparity between the two groups.

Using Pearson’s chi-square test of independence, we identified a statistically significant relationship between malnutrition and income poverty (*p* < 0.01), malnutrition and low level of education (*p* < 0.01), income poverty and low level of education (*p* < 0.01), low level of education and smoking (*p* < 0.05), malnutrition and smoking (*p* < 0.05), and smoking and daily alcohol consumption (*p* < 0.05).

## 4 Discussion

A basic limitation of our survey is the fact that it was an analysis of self-reported data, for which it is possible to assume, at least for part of the data, a conscious or unconscious distortion of the data by the respondents. For example, underreporting of alcohol consumption is well known (13). The population sample is representative, as it represents more than 2/3 of the notified cases of tuberculosis during the previous year. Another limitation is the association and mutual conditioning of some social determinants: e.g., alcoholism, smoking, and indoor air pollution are linked to household income. It can also be assumed that in the absence of active tuberculosis screening, cases from the most marginalised communities may have been missed. However, given the total size of this population, the number of such cases cannot be so large as to have a significant impact on the data.

We are aware that the questions we used do not cover all social aspects potentially affecting the incidence of tuberculosis. We deliberately limited ourselves to the questions used in the Eurostat survey. Such a selection guarantees that the questions are correctly formulated, validated, and the results of our investigation can be easily compared with the results of the investigation in the general population. We did not include, for example, self-reported ethnicity or race. We did not want to contribute to the segregation of ethnic minorities by publishing this data. In addition, a study from the United States of America demonstrated that socioeconomic factors are responsible for at least half of the increased risk of tuberculosis in ethnic and racial minorities (14). Immigration is known to have a significant impact on the incidence of tuberculosis (7). With the representation of nine subjects with a country of residence before 2020 other than Slovakia (8.8%) in our data, it is not relevant to determine the impact of immigration.

Despite the aforementioned limitations and the socioeconomic changes that have significantly shaped Slovakia in recent years, the results of our survey are in line with the knowledge known so far about the influence of social factors on the occurrence and course of tuberculosis. Several works have described the relationship between malnutrition and tuberculosis. Lönnroth et al., in an analysis of six of them, directly documented a mathematical relationship between body mass index (BMI) and the incidence of tuberculosis (15). A certain limitation of most studies investigating the relationship between nutritional status and tuberculosis is the fact that they are not based on the analysis of the entire population, but only of patients diagnosed with tuberculosis. It is therefore difficult to distinguish to what extent malnutrition is an independent risk factor or a consequence of the disease. This shortcoming was removed by a 7-year follow-up of a population of more than 11 million inhabitants of South Korea (16). This study also confirmed the inverse correlation between BMI and the incidence of tuberculosis, even after adjusting for age and sex; for smoking, alcohol consumption, physical activity and income; and for comorbidities (arterial hypertension, diabetes mellitus, and dyslipidemia). Body mass index as a significant risk factor for the development of tuberculosis is also one of the components of the composite risk score for the development of tuberculosis developed by Saunders et al. (17). Age, gender, and marital status affect the risk of tuberculosis. In a large Danish population analysis of tuberculosis patients, males aged 35–65 years living alone predominated (7). Similarly, an older North American study documented a more frequent occurrence in men, particularly in those over 70 years of age (18). A Slovak survey from 1997 documented the same gender distribution, with a predominance of men, as the present study (19). There was a slight shift to higher age categories in our data compared with the 1997 study. We did not observe any significant difference in the distribution of marital status, with the exception of the increase in the representation of the “widowed” category, compared to the data from 1997 (19). In agreement with this, the status “divorced” or “widowed” appears as a risk factor. Smoking as a risk factor for the development of tuberculosis was identified by many authors. In a meta-analysis of 33 relevant studies, the relative risk of TB disease in smokers versus non-smokers was reported to be 2.0 (20). In another meta-analysis based on 42 studies, this risk was reported at 2.3 (21). In a Portuguese study, the limit of the daily number of cigarettes smoked which significantly increased the risk of tuberculosis in men was determined to be greater than or equal to 20 per day (22). Smoking 20 or more cigarettes per day increased the odds for TB by 4.5 times. The length and intensity of smoking in our study did not change significantly compared to the Slovak survey 26 years ago (19). However, it should be noted that most of the data on the relationship between smoking and the incidence of tuberculosis comes from countries with low or middle income and a high incidence of tuberculosis. Excessive alcohol consumption is considered one of the risk factors for the development and progression of tuberculosis. A meta-analysis of 36 relevant studies confirmed not only a significantly higher incidence of tuberculosis in people who regularly consume alcohol, but also a relationship between the risk of the disease and the dose of alcohol (23). A review of several studies on this topic has shown that the risk of tuberculosis increases significantly with average daily alcohol consumption exceeding 40 g (24). Compared to the general population, our tuberculosis patients more often admitted to daily alcohol consumption. Paradoxically, they less often reported alcohol consumption in the categories “monthly or less” and “2 to 3 times a month.” Moreover, they more often reported that they had not drunk alcohol in the last year or had never drunk it. Similarly, our survey contains some surprising data on the frequency of heavy episodic drinking. Our tuberculosis patients more often admitted to drinking more than 6 standard alcoholic drinks on a single occasion weekly or monthly. However, the proportion of tuberculosis patients who declared that they had never drunk such a quantity of alcohol on a single occasion was higher compared to the general population. Since excessive alcohol consumption is not socially accepted, there is a known tendency to distort data on its consumption in self-reporting (13). This distortion should theoretically be the same in tuberculosis patients as in the general population. However, we can assume that people with a disease with a widely known relationship to alcohol consumption may have a stronger inhibition against admitting actual consumption. Moreover, neither the frequency of alcohol consumption nor the frequency of heavy episodic drinking may reflect the total amount consumed. The effect of overcrowding has been highlighted by a Canadian and New Zealand study (25, 26). Compared to the study of tuberculosis patients in Slovakia in 1997, the number of people living in one household had decreased (19) in our TB cohort. The average number of people per living room in our survey was 1.17 ± 0.93, a value greater than 1, which is considered to be indicator of overcrowding, was exceeded in 32 respondents (31.4%). A low level of education in tuberculosis patients has been reported by Nordholm et al. (7). In a Romanian case–control study, illiteracy was one of the identified independent risks for tuberculosis (27). In a survey of the Slovak population of tuberculosis patients, the representation of patients with basic education was the same as in our work, but the proportion of people without any completed education in our study is lower than in 1997 (19). Unemployment has decreased in the population of Slovak tuberculosis patients compared to the situation from 1997 (19). Nevertheless, it was significantly more frequent than in general population. Poverty as a risk factor for tuberculosis has been investigated in various ways. A Romanian questionnaire survey identified low household income as the second strongest risk factor for tuberculosis (27). A low income or dependence on social benefits was described in a large Danish study (7). Poverty also appears to be a risk factor in the North American population (18). Slovakia is among the countries with the lowest income inequalities (28). Direct economic indicators and related material conditions are therefore not as strong risk factors for tuberculosis as in countries with low average incomes and high income inequality (some countries in South America, Asia, and Africa), from which there are reports of high tuberculosis risk associated with low household income.

We found significant differences in the distribution of all investigated social risk factors between the general population and TB patients in Slovakia. Moreover, a significant trend in the proportions was identified across the categories of the contingency tables for all ordinal variables with more than two levels. Our results confirm that even today in Slovakia, despite a very high HDI and a low incidence of tuberculosis, this disease is still a disease of people with low social status.

Interventions to influence the social determinants of tuberculosis were not the subject of our work. However, our findings confirm that such measures are also necessary in Slovakia and point to the highest risk factors that should be targeted. Moreover, they are an incentive for considering the reintroduction of an active tuberculosis screening program in the high-risk population.

## Data Availability

The raw data supporting the conclusions of this article will be made available by the authors without undue reservation.
